# Facile emulsion mediated synthesis of phase-pure diopside nanoparticles

**DOI:** 10.1038/s41598-018-21485-9

**Published:** 2018-02-15

**Authors:** Elena Tajuelo Rodriguez, Lawrence M. Anovitz, Caleb D. Clement, Adam J. Rondinone, Michael C. Cheshire

**Affiliations:** 1 Fusion and Materials for Nuclear Systems Division, MS 6148, P.O. Box 2008, Bldg. 4500S, Oak Ridge National Laboratory, Oak Ridge, TN 37831-6148 USA; 2 Chemical Sciences Division, MS 6110, P.O. BOX 2008, Bldg. 4100, Oak Ridge National Laboratory, Oak Ridge, TN 37831-6110 USA; 3 Center for Nanophase Materials Science Division, MS 6493, P.O. BOX 2008, Bldg. 8600, Oak Ridge National Laboratory, Oak Ridge, TN 37831-6493 USA

## Abstract

Diopside is a common natural pyroxene that is rarely found in a pure state, since magnesium is often partially substituted by iron, and other elements (sodium and aluminum) are often present. This pyroxene, along with feldspars and olivines, is common in concrete. As the prospective license renewal of light water reactors to 80 years of operation has raised concerns on the effects of radiation in the concrete biological shield surrounding the reactors, mineral nanoparticles can be valuable to perform amorphization studies to inform predictive models of mechanical properties of irradiated concrete. The synthesis of diopside nanoparticles was achieved in this study using a reverse-micelle sol-gel method employing TEOS, calcium chloride and Mg(MeO)_2_ in a methanol/toluene solution. Tert-butylamine and water were used as hydrolysis agents, and dodecylamine as a surfactant. The resulting amorphous precursor was centrifuged to remove organics and fired at 800 °C. Additional reaction with hydrogen peroxide was used to remove amine remnants. TEM and SEM examinations revealed a product comprised of 50–100 nm diameter nanoparticles. XRD indicated phase pure diopside and BET indicated a surface area of 63.5 m^2^/g before peroxide treatment, which at a bulk density of 3.4 g/cm^3^ is equivalent to particles with diameter of 28 nm.

## Introduction

The pyroxene family is an extremely important and well-known group of rock-forming minerals, occurring in igneous and metamorphic rocks^1^, as well as lunar and meteoritic materials^2^. Because of this, mineralogical and geochemical studies have been ongoing for many years. For instance, the structure of diopside was determined very early by Warren and Bragg^3^, just fifteen years after the crystal structure determination of diamond^4^, and group I halide salts^5^. Pyroxenes form an important part of Bowen’s reaction series^6^, and define the onset of granulite metamorphism^7,8^.

Despite the large number of mineralogical and geochemical studies on the pyroxenes, significant questions remain particularly with respect to surface chemistry. For many minerals, the analysis of nanoparticles and other surfaces has shown that the surface properties may vary significantly from those ascribed to the bulk^9–14^. No studies of these effects in pyroxenes are available. Similarly, the effects of radiation on the structural properties of pyroxenes are unknown. However, the foreseen extension of the license for Light Water Reactors in the US to 80 years of operation has resulted in a series of questions regarding the effect of irradiation on the concrete biological shield that surrounds the reactors. As the biological shield has both structural and radiation protection functions, it is important to ensure its durability and safety for the proposed extended life cycle, but the mechanical properties of concrete (tensile and compressive strength) deteriorate with exposure to high neutron doses (>10^19^ n/cm^2^)^15^. The underlying cause of this loss in mechanical properties is RIVE (radiation induced volumetric expansion)^15^. Minerals such as quartz, feldspars, olivines and pyroxenes are commonly found in concrete aggregates. These silicates exhibit low resistance to neutron irradiation and suffer from RIVE, which ultimately causes cracks in the aggregates and strain in the cement paste. Data on volumetric expansion with dose for a variety of minerals is, therefore, critically needed in assessing reactor safety.

Amorphization is another process known to occur during irradiation that affects the structural properties of minerals. Thus, the determination of amorphization rates and critical amorphization doses is central to assessing the influence of irradiation on aggregates and the risk of radiation-induced damage. Ideally, neutron irradiation experiments should be performed to obtain critical amorphization doses and volumetric expansion. However, neutron irradiation in test reactors can be time consuming and costly. Ion-beam experiments, however, have been shown to provide a legitimate alternative. Eby, for instance, studied the critical amorphization ion doses of several silicate minerals by obtaining diffraction patterns of samples bombarded with 1.5 MeV Kr^+^ ^16^. This technique has been shown to be successful. However, to date only limited information is available regarding the amorphization rate of minerals with ion dose. In addition, further experiments by Harbsmeier and Bolse^17^ indicated that the amorphization doses in Eby’s work may have been overestimated. Harbsmeier and Bolse^17^ found that complete amorphization of α-quartz occurred at a dpa (displacement per atom) of 0.04, whereas Eby’s data suggested a much larger dpa of 0.11 was required.

One very convenient method to perform ion-beam amorphization studies utilizes mineral nanoparticles in coupled, *in-situ*, ion beam/TEM (Transmission Electron Microscope) experiments. This has been done for several systems such as Si^18^, rutile, brookite, anatase, cassiterite^19^, olivine, enstatite, troilite and pyrrhoite^20^. Calculations of the associated damage dpa using codes such as SRIM^21^ can be used to correlate the damage to an equivalent neutron dose for the same dpa. Volumetric expansion information can be easily obtained from ion-beam experiments with mineral thin sections, and obtaining amorphization rates from *in-situ* TEM of nanoparticles is much faster than preparing TEM samples from the neutron-bombarded, and potentially radioactive, thin sections. This method however, requires the availability of suitable mineral nanoparticles. Thus, development of methods for the synthesis of mineral nanoparticles is an important step in advancing our knowledge in amorphization processes, and improving recently developed physics-based predictive models for changes in the mechanical properties of concrete as a function of neutron fluence^22–25^. Synthetic materials have several advantages over natural minerals for this application. Natural materials can be difficult to purify, often contain numerous chemical solid solutions, are difficult to grind into nanoparticles without significant structural damage, and the presence of natural radioactive inclusions, such as alpha emitters, can induce structural changes not accounted for in the experiment. In particular, the development of successful methods to produce diopside nanoparticles can have an impact not only on amorphization studies, but on other disciplines. These nanomaterials may have many potential uses since they are bio-compatible for dental fillings, and can also serve to study fluid sorption and silicate surface properties. This paper describes a relatively simple method for the synthesis of such materials.

## Previous Work

The synthesis of diopside has been reported in the literature using a wide range of methods. Nonami *et al*.^26^ synthesized diopside by a sol-gel method using metal alkoxides followed by crystallization in air at 840 °C. Iwata *et al*.^27^ modified this method using a metal alkoxide and metal salts. In this case pure diopside was only obtained after sintering pellets to 1100 °C for 2 hours. Hayashi *et al*.^28^ synthesized micrometer sized diopside (2–8.5 μm particle size) via several sintering processes. Some of these employed amorphous silica, calcium carbonate, magnesium hydroxide and a mixture of additives as raw components. In one route TEOS, calcium nitrate hydrate and magnesium nitrate hydrate were mixed in an ethanol solution, to which ammonium hydroxide was added to precipitate amorphous material. Crystallization of diopside occurred at temperatures from 830 °C to 1275 °C, depending on the route and precursors, as indicated by thermogravimetric data.

Hayashi *et al*.^29^ used a variety of solution routes to synthesize diopside: coprecipitation, homogeneous precipitation and a sol-gel method. The starting solution for the three paths was comprised of calcium nitrate hydrate, magnesium nitrate hydrate and TEOS in ethanol. Ammonium hydroxide was added in the coprecipitation method, and urea for homogenous precipitation. After firing the three products at 1300 °C for 2 hours, only the coprecipitation method yielded a pure product. Considerable amounts of akermanite and merwinite were found in the products of the other two approaches. The three samples were reported to have particle sizes in the sub-micrometer scale (0.1–0.5 μm and larger). Kumar *et al*.^30^ synthesized diopside via a sol-gel method using magnesium nitrate, calcium nitrate and rice straw. Calcined rice straw was added to citric acid and stirred while boiling, then the magnesium and calcium sources were added. Stirring was continued until a gel was obtained. This gel was dried at 100 °C, ground and calcinated at 1050 °C for 2 h. The final diameter of the particles was 0.7 μm.

A sol-gel method for the synthesis of diopside was employed by Iwata *et al*.^31^ using calcium nitrate hydrate, magnesium nitrate hydrate and TEOS as main reactants in an ethanol/water solution. Ammonium hydroxide was introduced to the solution to force precipitation. After heating at 850 °C their product was still not fully crystallized. Full crystallization to diopside required heating the precipitates to 1100 °C. Gorbhanian *et al*.^32^ used a similar procedure, incorporating other steps in the thermal treatment, followed by high energy ball milling to obtain nanoparticles with sizes ranging from 35 to 65 nm. Choudhary *et al*.^33^ synthesized diopside by a sol gel method adding nitric acid to a solution containing magnesium nitrate, L-alanine, eggshell solution, and TEOS. The solution was heated at 50 °C for 1 hour, dried in an oven at 150 °C for 4 hours, decomposed at 400 °C for 2 hours in a preheated muffle furnace, and calcined at 850 °C for 6 hours. The resulting product looked porous, but neither the particle size nor the surface area was reported. Salahinejad and Vahedifard^34^ obtained pure diopside nanoparticles with an average size of 70 nm employing a sol-gel method with silicon tetrachloride, magnesium chloride and calcium chloride as precursors. Ethanol was used to prepare the solution, and an aqueous ammonia solution was used as a precipitation agent. The creamy precipitates were calcined at 700 °C. The surface area of the final product was not reported. Kazemi *et al*.^35^ synthesized diopside nanoparticles by grinding eggshell powder, SiO_2_ and MgO in a planetary ball mill for 6 h. The ground powder was then pressed in a pellet and sintered at 1200 °C for 3 h. The nanoparticles produced had a diameter of ~50 nm. Ghomi *et al*.^36^ synthesized nanodiopside by a sol-gel method dissolving Ca(NO_3_)_2_·4H_2_O, MgCl_2_·6H_2_O and TEOS in ethanol which was stirred to form a solution. The dried gel was then calcined for 2 h at 800 °C and then milled for 10 h. The nanoparticles formed were less than 50 nm in diameter. Razavi *et al*.^37^ prepared diopside nanoparticles by dissolving Ca(NO_3_)_2_·4H_2_O, MgCl_2_·6H_2_O and Si(OC_2_H_5_)_4_ in ethanol. The gel that formed after stirring this solution was dried at 100 °C for 24 h and then calcined at 700 °C for 2 h. After calcination the powder was ball milled for 10 h. The resulting nanoparticles were 50–100 nm in diameter, were agglomerated, and had irregular shapes.

Most of the synthesis routes discussed in this section yielded particles on the micrometer scale and employed sintering and calcination temperatures >800 °C. To obtain nanoparticles, high energy milling was often employed. The objective of the work presented in this publication, therefore, was to implement a simple synthesis procedure minimizing the use of additional mechanical treatments such as ball milling, and using relatively low temperatures, to yield nano-sized diopside with a high surface area suitable for amorphization studies, as well as analysis of its surface properties. A sol-gel approach mediated by a reverse-micelle micro-emulsion was used. Particle growth is self-limited in emulsions, promoting the formation of nanoparticles. In the present approach, the alkoxides, TEOS and Mg(MeO)_2_ were dissolved in toluene, while calcium nitrate was dissolved in methanol. The reaction was then likely limited by the diffusion of alkoxides into the emulsion, thus avoiding the poorly controlled aggregation and large variation of stoichiometry that is common for other approaches^28,29,37^.

## Results

### XRD

**Figure 1:**
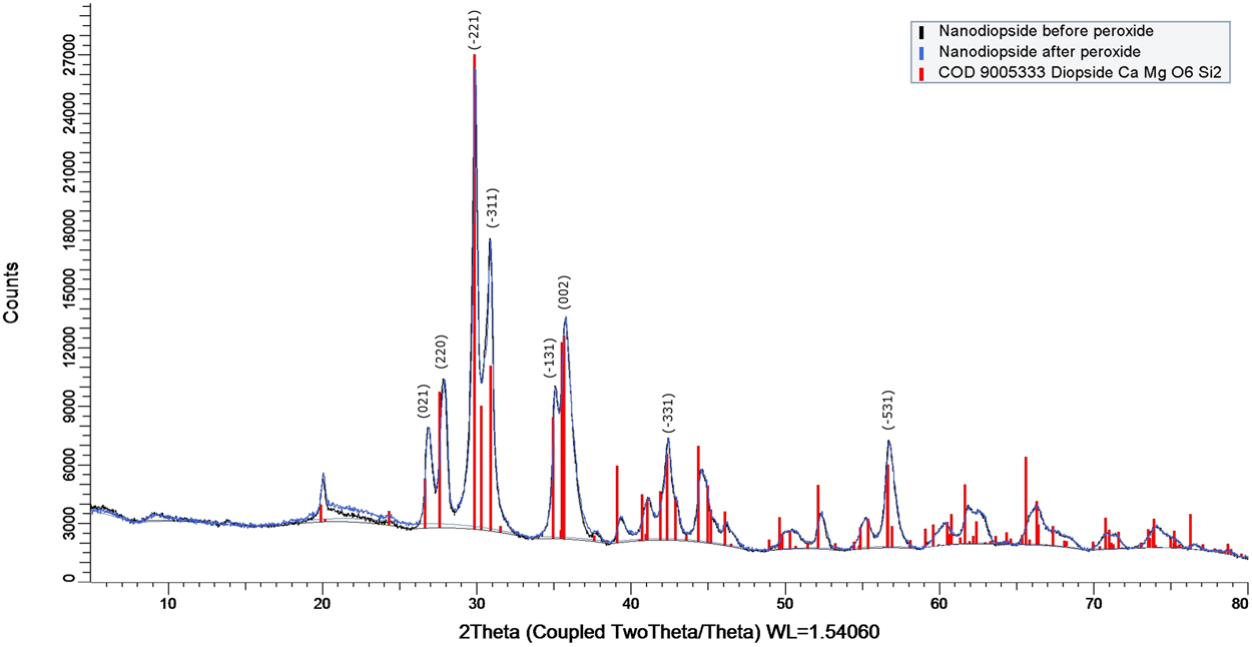
X-ray diffraction patterns of the synthetic nanoparticles of diopside before (black) and after (blue) reaction with hydrogen peroxide. A reference pattern of diopside (red lines) was taken from the Chemical Open Database.

**Figure 2:**
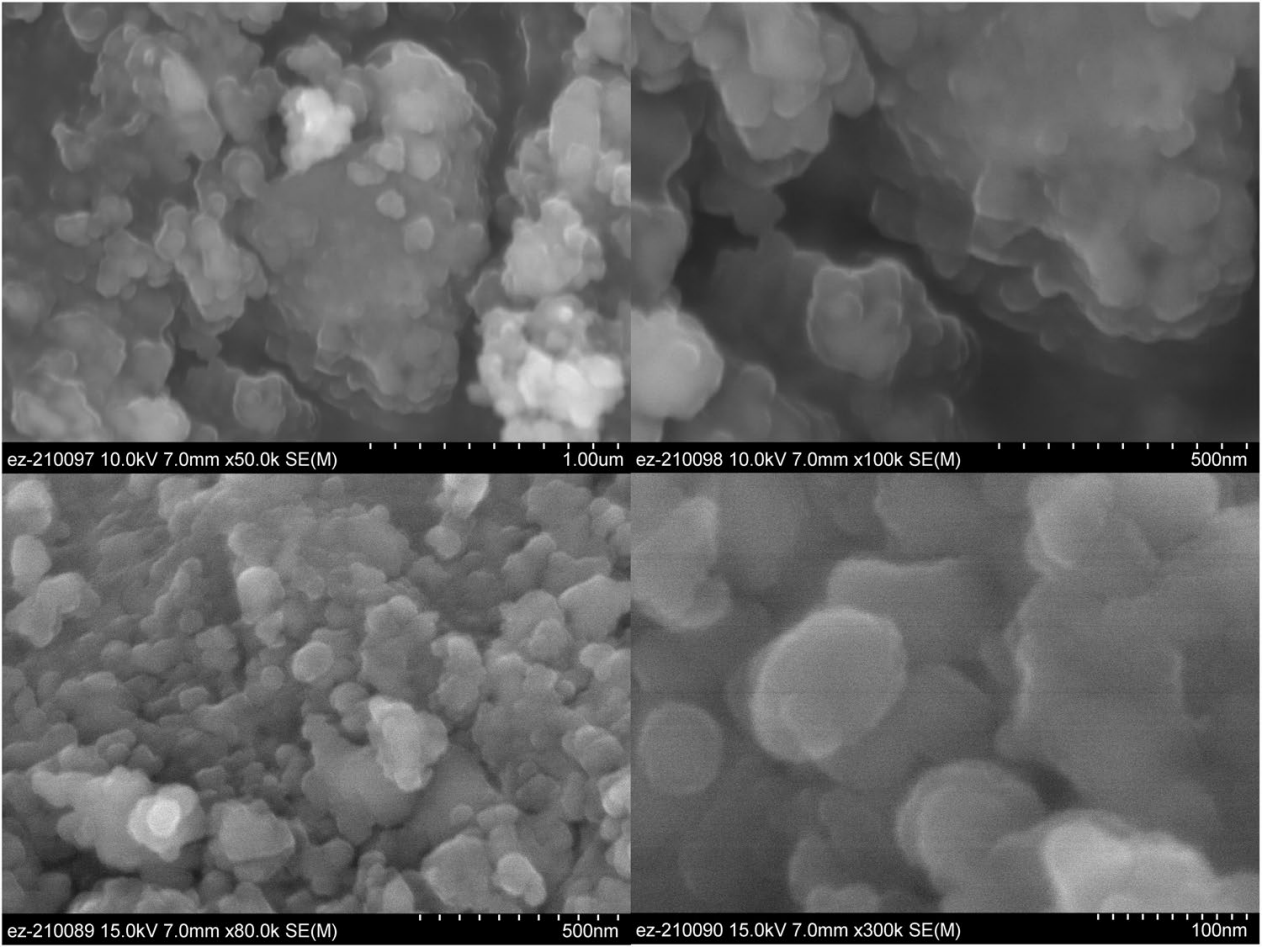
Secondary electron images of synthetic diopside before (upper row) and after (lower row) treatment with hydrogen peroxide.

The XRD patterns obtained before/after reaction with hydrogen peroxide are shown in Fig. 1. All peaks in the patterns could be identified as diopside, confirming the purity of the crystalline synthetic product. There are almost no differences in the patterns before/after treatment. The unit cell parameters given by Rietveld refinement for the sample before peroxide treatment were: a = 9.7375 ± 0.0007 Å, b = 8.9029 ± 0.0006 Å, c = 5.2664 ± 0.0003 Å in the monoclinic space group B2/b, with R_Bragg_ being 5.18, goodness of fit (χ) of 5.96 and a micro strain of 0.28%. The refinement of the pattern from the diopside sample after peroxide treatment yielded the following unit cell parameters: a = 9.738 ± 0.001 Å, b = 8.9020 ± 0.0006 Å, c = 5.2654 ± 0.0004 Å with an R_Bragg_ value of 3.86, goodness of fit (χ) of 5.65 and a micro strain of 0.28%, using the same monoclinic space group as for the previous pattern. Both refinements yielded an estimated mean crystal grain diameter of ~22 nm, which compares to the 50 nm crystallite size obtained by Salahinejad and Vahedifard^34^ using their most intense diffraction peak. The crystallite size is expected to coincide with the physical diameter of the particles in the case where the individual particles consist of a single crystalline domain, which suggests that our particles are polycrystalline since their diameter is in the range of 50 to 100 nm (Fig. 2).

### BET

The BET analysis of the sample before peroxide treatment yielded a surface area of 63.5 m^2^/g. No uncertainties are provided for the surface area since only one data set was analyzed. Assuming spherical particles and a density of 3.4 g/cm^3^ ^38^, this yields a particle size of 28 nm. This is in good agreement with the crystallite size obtained through Rietveld refinement of the diffraction pattern of the same sample. The surface area obtained after peroxide treatment was 50.5 m^2^/g corresponding to a particle size of 35 nm, slightly higher than the value obtained by Rietveld refinement. The decrease in surface area may indicate aggregation that was previously inhibited by the surfactants, and suggests that keeping the surfactants in place may be beneficial under some circumstances. Interestingly, this was observed before for similarly synthesized nanoenstatite, while the opposite case was observed for nanoforsterite^39^.

### SEM/TEM

**Figure 3:**
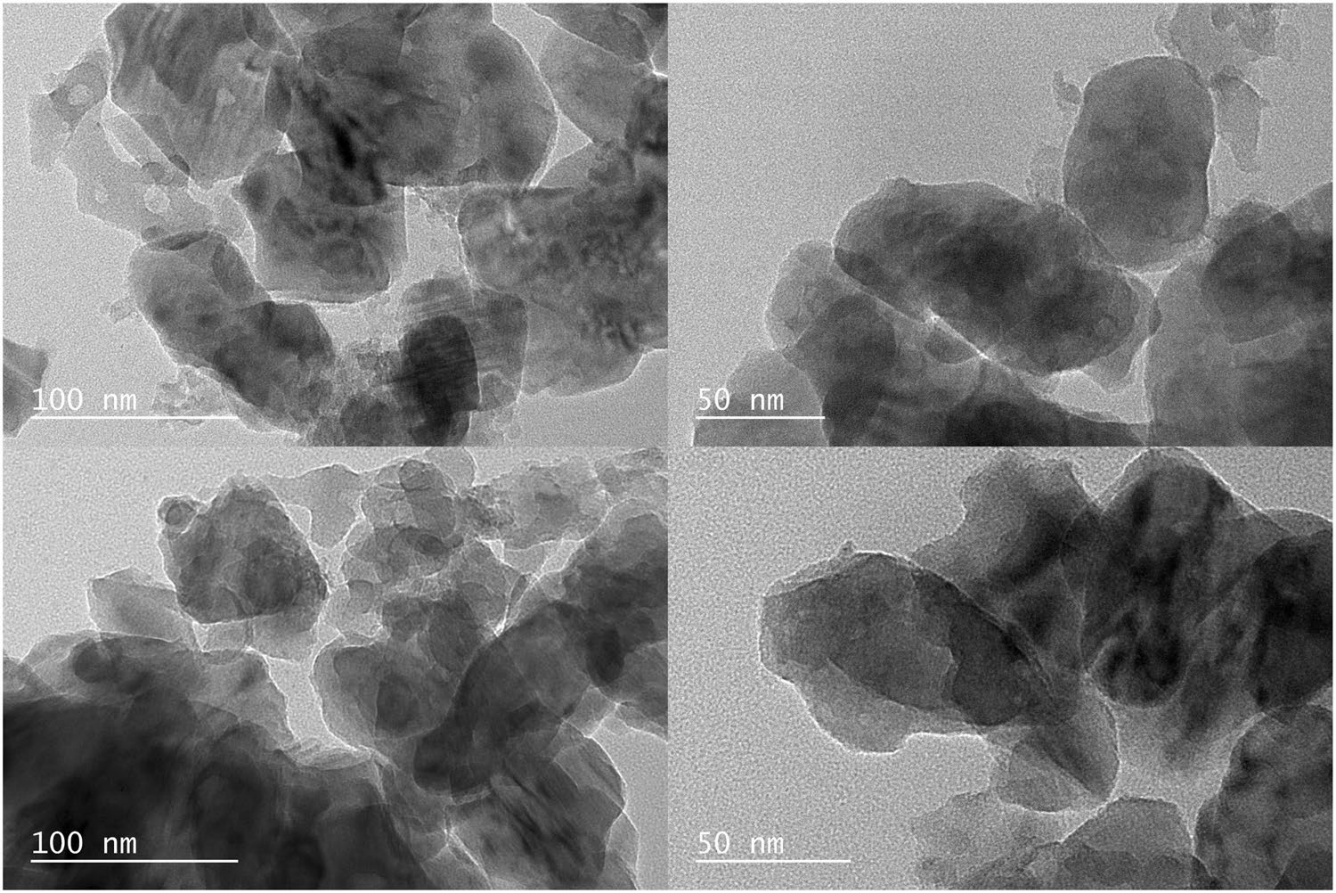
TEM bright field images of synthetic diopside nanoparticles before and after reaction with hydrogen peroxide (upper and lower row respectively). Organic surfactants can be seen on the surfaces of some of the particles and some crystalline facets can be observed.

Secondary electron images of the sample before and after peroxide treatment are shown in Fig. 2. The micrographs show particles of 50–100 nm diameter agglomerated in larger, rounded objects, stacked to form yet larger particles. This was observed for samples both before and after treatment with peroxide. This agglomeration is typical for nanoparticles synthesized in a similar manner, and has been noted before for nanoenstatite and nanoforsterite^39^. TEM micrographs in Fig. 3 show a similar average particle size of 50 to 100 nm, although smaller particles are also present. What appears to be organic residue is visible on the surface of some of both the peroxide-untreated and treated particles. The observed average particle size is larger than the value of the crystallite size determined by Rietveld refinement and the particle size given by BET. In the case of XRD, line broadening is related to the number of adjacent crystalline planes. Therefore, the particle size obtained through Rietveld refinement correlates with the crystallite size and not to the actual physical particle size. In fact, the TEM images also show crystalline domains or facets on the order of 20–30 nm, a size that coincides with the value given by Rietveld refinement. There may also be size discrepancies due to the fact that both the Rietveld refinement and BET analyses assume the particles are spherical. It is evident from the TEM micrographs that particles are not spherical, since they mainly have elongated shapes and present sharp edges in some cases.

## Discussion

The results presented in this paper demonstrate that the sol-gel synthesis method previously developed by Anovitz *et al*.^39^ to obtain nano-scale forsterite and enstatite, and by DeAngelis *et al*.^40^ to obtain fayalite, can be easily modified to produce clino- as well as orthopyroxenes. XRD, SEM, and TEM examination indicated that pure diopside was synthesized, as no evidence of the presence of any other silicate or oxide phases was found. The synthesis route presented is a very simple procedure that requires firing temperatures of only 800 °C, and yields nanosized materials with a reasonably large surface area without the need of high energy mechanical grinding. The success of the synthesis approach was attributed to the use of a micro-emulsion, which limits particle growth, and to the fact that the alkoxides, TEOS and Mg(MeO)_2_ were dissolved in toluene, while calcium nitrate was dissolved in methanol, likely limiting the reaction by the diffusion of alkoxides into the emulsion. This lead to a control of stoichiometry and particle aggregation.

## Methods

### Synthesis method

This synthesis was performed utilizing a modification of the method originally developed by Anovitz *et al*.^39^ for the synthesis of forsterite and enstatite. Sources of magnesium, calcium and silica were magnesium methoxide (Mg(MeO)_2_) (Alfa Aesar), calcium chloride (Sigma Aldrich, Reagent Plus, >99%) dehydrated at 210 °C for 2 hours under vacuum and stored in a glove box, and tetraethylorthosilicate (TEOS) (Acros Organics) respectively. Prior to synthesis, the concentration of magnesium methoxide was analyzed in triplicate by hydrolyzing small amounts of the solution with concentrated HCl. The hydrolyzed solution was dried and fired in air at 800 °C overnight. By comparing the weight of the resultant MgO to the original weight of Mg(MeO)_2_, the concentration of MgO in solution was calculated as approximately 6.206·10^−4^ mol MgO per gram of Mg(MeO)_2_ (~1.5 wt%). The composition of the TEOS was previously analyzed using a similar approach, and was found to be close to the nominal value^39^.

Synthesis of diopside was performed in a 250-mL 3-neck flask attached to a glass condenser connected to a Schlenk line. The condenser was connected to a recirculating water bath at 18 °C. The flask was placed in a silicone oil bath on top of a magnetic stirrer/hotplate. The flask was dried by alternately purging with argon gas and evacuating using a roughing pump several times. Argon remained flowing during the experiment (as checked with a bubbler at the exit) to limit air access during addition of the starting materials.

To begin the synthesis 82-mL of toluene (Fischer Scientific) and 22-mL of methanol (Fischer Scientific) were added to the flask, along with a stir bar. These were stirred for five minutes to homogenize the mix. 43.75 g of Mg(MeO)_2_ was then added to the homogenized solution, along with stoichiometric masses (according to diopside composition, CaMgSi_2_O_6_) of TEOS first, and then calcium chloride in methanol. The latter solution was prepared by combining anhydrous calcium chloride and 22 mL of anhydrous methanol (Sigma Aldrich) in a glovebox to minimize exposure to water. The beaker containing this solution was then covered with parafilm to isolate it from the atmosphere, removed from the glovebox, and transported to the fumehood containing the Schlenk line. Upon introduction of the calcium chloride solution, the contents of the 3-neck flask turned cloudy. The mixture was then left stirring for five minutes. 0.182 g of tert-butylamine (Sigma Aldrich) and 0.910 g of melted dodecylamine (Sigma Aldrich) were mixed in 25-mL of toluene and then added to the flask, which was capped and allowed to stir for five minutes. This amine solution was needed to act as surfactant/hydrolysis agents.

Once the solution had been prepared, the hot plate under the silicone oil bath was set to 230 °C to bring it to reflux while the solution was stirred continuously under a constant Ar flow. The system was then refluxed for approximately 4 hours. After this time, the heat was turned off and approximately 12-mL of DI water (from a Millipore system yielding water with a resistivity of at least 18 MΩ cm) were added in approximately 1 mL increments to ensure complete hydrolysis until no further hydrolyzing reaction was observed. Upon water addition, a milky white gel formed immediately in a non-uniform pattern which homogenized on stirring. The solution was then left to cool while stirring overnight.

After cooling, the solution had expanded to a white, fluffy gel. This was transferred from the three-neck flask to several centrifuge tubes. Additional methanol and toluene were used to wash the flask to transfer all contents to the tubes. The product was centrifuged, decanted and washed with a toluene/methanol mix four times to remove most of the solvents and surfactants using the following procedure: The centrifuge tubes were placed in an Eppendorf multipurpose centrifuge for 1 hour at 6000–7000 rpm. The solvent was then decanted from the top of the tubes, and a solution of 50% methanol- 50% toluene was added to the remaining gel. The product was resuspended using an ultrasonic probe and a Vortex mixer, and the tubes were then returned to the centrifuge. After the second washing cycle, the product changed from a white fluffy gel to a transparent, thick gel, and became more difficult to resuspend. Upon completion of the washing cycles, the samples were dried in a fume hood for 3 days, then transferred to crucibles and dried at 70 °C for 3 days. The dried product was then fired at 800 °C overnight, and ground to a fine powder with a mortar and pestle. The final product was then reacted with a 30% H_2_O_2_ solution at room temperature overnight to remove any remaining amine. A stir bar was then added to the solution and it was agitated for 2 hours at 60 °C. Finally, the sample was dried at 70 °C under vacuum for 3 days.

### Characterization methods

X-ray diffraction analysis (XRD) was performed using a Bruker D2-Phaser desktop X-ray diffractometer operating at 30 kV and 10 mA with a CuKα source. The scans were acquired using a 5–80° 2θ range, with a step size of 0.03° and a time per step of 3 s. 1 mm incident and diffracted beam slits were used, as was a 3 mm anti-scatter slit. The powder was packed into a holder on a zero-background silicon plate. The patterns were compared with standards using DIFFRAC.EVA^TM^ software that includes a pattern list from the Chemical Open Database. The patterns were analyzed with High Score Plus^TM^ for Rietveld refinement.

Scanning electron microscope (SEM) images were obtained using a Hitachi S-4800 at 10 kV and 15 kV in secondary electron mode. A small amount of powder was deposited on carbon tape placed on an SEM stub mount. The mount was coated with a carbon film ~10 nm thick using a Cressington 208 carbon coater. TEM bright field images were obtained using a JEOL JEM-2100F FEG-S/TEM operating at 200 kV and magnifications of 80–120 kx. A small amount of the final powder was sonicated for 2 min in ethanol and a few drops of the solution were then deposited over a Cu grid with a carbon film.

The specific surface area of the final product was obtained using a Belsorp-MAX (MicrotracBEL Corp) Brunauer-Emmett-Teller (BET) analyzer. Nitrogen gas was used as the adsorbate and liquid nitrogen as the cryogen. Eleven pressure points were obtained, and the surface area calculated using the standard BET equation^41^. The particle size in nm was estimated using the equation: PS = 6000/ (BET surface area m^2^/g · density g/cm^3^).

## Electronic supplementary material

Supplementary Dataset 1

Supplementary Dataset 2
