# Author Correction: Facile emulsion mediated synthesis of phase-pure diopside nanoparticles

**DOI:** 10.1038/s41598-018-24110-x

**Published:** 2018-04-18

**Authors:** Elena Tajuelo Rodriguez, Lawrence M. Anovitz, Caleb D. Clement, Adam J. Rondinone, Michael C. Cheshire

**Affiliations:** 1 Fusion and Materials for Nuclear Systems Division, MS 6148, P.O. Box 2008, Bldg. 4500S, Oak Ridge National Laboratory, Oak Ridge, TN 37831-6148 USA; 2 Chemical Sciences Division, MS 6110, P.O. BOX 2008, Bldg. 4100, Oak Ridge National Laboratory, Oak Ridge, TN 37831-6110 USA; 3 Center for Nanophase Materials Science Division, MS 6493, P.O. BOX 2008, Bldg. 8600, Oak Ridge National Laboratory, Oak Ridge, TN 37831-6493 USA

Correction to: *Scientific Reports* 10.1038/s41598-018-21485-9, published online 15 February 2018

The Acknowledgements section in this Article is incomplete.

“L.M.A. and M.C.C. were supported by the Division of Chemical Sciences, Geosciences, and Biosciences, Office of Basic Energy Sciences, U.S. Department of Energy.”

should read:

“L.M.A. and M.C.C. were supported by U.S. Department of Energy, Office of Science, Office of Basic Energy Sciences, Chemical Sciences, Geosciences, and Biosciences Division.”

